# Insights into the m^6^A demethylases FTO and ALKBH5 : structural, biological function, and inhibitor development

**DOI:** 10.1186/s13578-024-01286-6

**Published:** 2024-08-27

**Authors:** Zewei Gao, Xuan Zha, Min Li, Xueli Xia, Shengjun Wang

**Affiliations:** 1 Department of Laboratory Medicine，Jiangsu Province Engineering Research Center for Precise Diagnosis and Treatment of Inflammatory Diseases, The Affiliated Hospital of Jiangsu University, Zhenjiang, 212001 China; 2 Department of Immunology, Jiangsu Key Laboratory of Laboratory Medicine, School of Medicine, Jiangsu University, Zhenjiang, China; 3 Department of Laboratory Medicine, Affiliated People’s Hospital, Jiangsu University, Zhenjiang, 212002 China

**Keywords:** N6-methyladenosine (m6A), Demethylases, FTO, ALKBH5

## Abstract

N6-methyladenosine (m^6^A) is dynamically regulated by methyltransferases (termed “writers”) and demethylases (referred to as “erasers”), facilitating a reversible modulation. Changes in m^6^A levels significantly influence cellular functions, such as RNA export from the nucleus, mRNA metabolism, protein synthesis, and RNA splicing. They are intricately associated with a spectrum of pathologies. Moreover, dysregulation of m^6^A modulation has emerged as a promising therapeutic target across many diseases. m^6^A plays a pivotal role in controlling vital downstream molecules and critical biological pathways, contributing to the pathogenesis and evolution of numerous conditions. This review provides an overview of m^6^A demethylases, explicitly detailing the structural and functional characteristics of FTO and ALKBH5. Additionally, we explore their distinct involvement in various diseases, examine factors regulating their expression, and discuss the progress in inhibitor development.

## Background

**Fig. 1:**
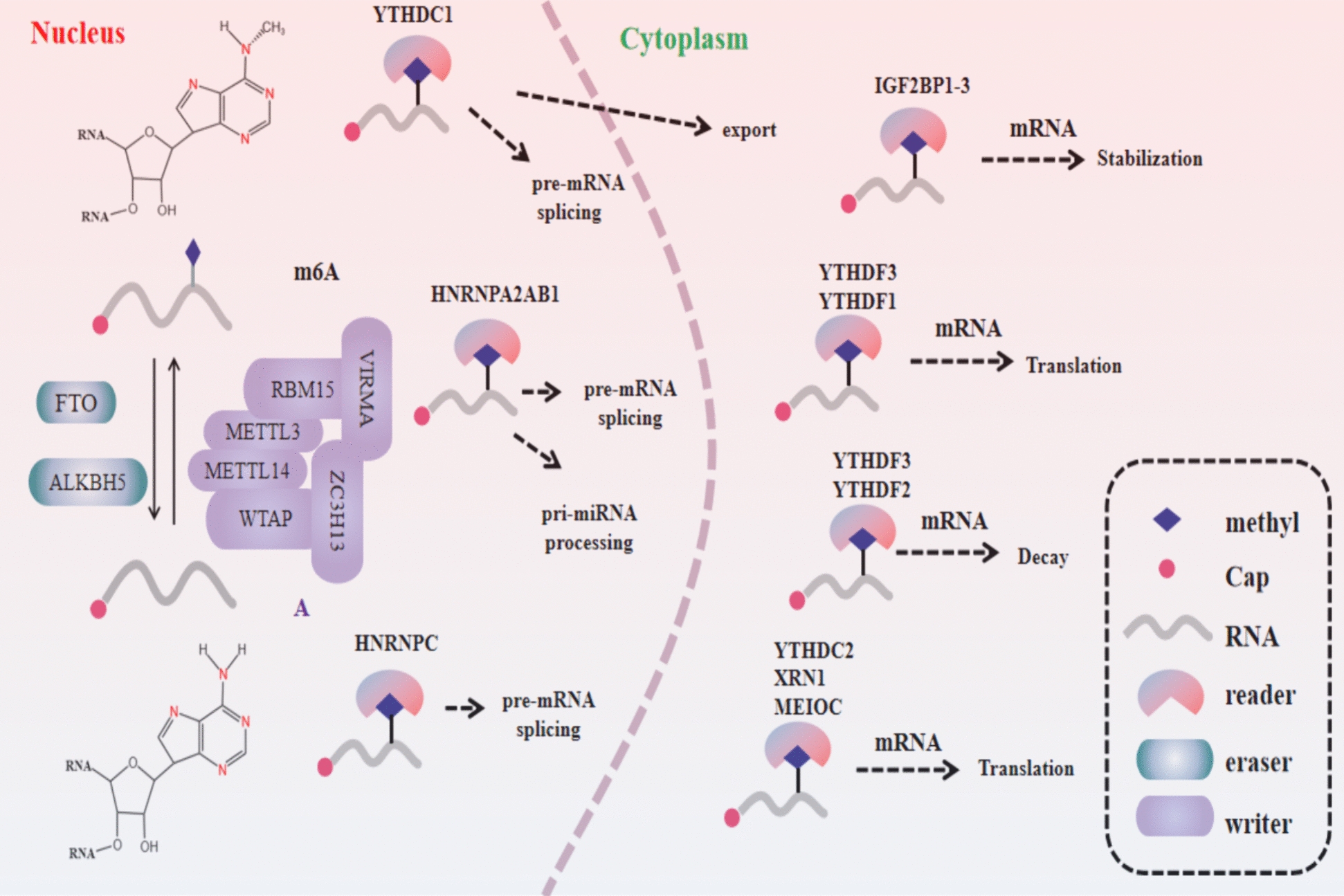
The dynamic process of m6A modification and its regulatory role. The m6A modification protein complex mainly consists of writers, erasers, and reader proteins. Among them, METTL3 serves as the core protein responsible for exerting the m6A modification function. The erasers include the two discovered demethylases FTO and ALKBH5, which play a role in demethylation. Reader proteins consist of various families such as the YTHDF family, YTHDC family, and IGF2BP family, which recognize specific m6A methylations. These reader proteins recruit downstream effector molecules, thereby influencing RNA stability, translation, transcription, RNA-RNA interactions, and other processes related to RNA metabolism

 Over a hundred chemical alterations in cellular RNA have been cataloged, with N6-methyladenosine (m^6^A) modification standing out as the most prevalent and evolutionarily conserved post-transcriptional modification in eukaryotic organisms, particularly in advanced eukaryotes [1, 2]. The m6A modification is methylation located on the sixth nitrogen atom of adenosine [3]. As one of eukaryotic RNA’s most common chemical modifications, m6A plays crucial roles in RNA stability, localization, translation, splicing, and transportation [4]. The m6A modifications are highly conserved in different species, especially in the testis and liver organs. The m6A modifications undergo dynamic, reversible changes in different tissues, developmental stages, and physiological processes [5]. Disruption or disturbance of this dynamic modification may lead to dysregulation of cellular regulatory mechanisms, thereby causing various diseases. The process of RNA m6A methylation is dynamically and reversibly regulated by m6Amethyltransferases, demethylases, and binding proteins, referred to as “writers,” “erasers,” and “readers,” respectively (Fig. 1) [6]. The m6A methyltransferase complex (MTC) is involved in the regulation of the methylation process of m6A. It consists of three core components (METTL3, METTL14 and WTAP) and four regulatory subunits (VIRMA, ZC3H13, HAKAI and RBM15) involved in the methylation process. The erasers responsible for demethylation include FTO and ALKBH5. Specific reader proteins for m6A methylation, such as IGF2BP1/2/3, YTHDF1/2/3, and YTHDC1/2, interpret and functionally regulate m6A modification. These proteins collectively participate in RNA m6A methylation and demethylation, playing crucial roles in gene expression and cellular functions [7]. The methyl group catalyzed by METTL3 can be recognized and bound by “reader” proteins such as YTHDC1/2, YTHDF1-3, and IGF2BP1-3, specifically recognizing m6A-modified transcripts [8]. Recognition of different methylated sites by distinct reader proteins leads to varied biological responses, such as regulation of RNA translation and stability [9]. Individual m6A reader proteins may exhibit redundant or specific functions in different cellular contexts [1]. M6A-binding proteins typically feature YT521-B homology (YTH) domains, crucial for interacting with m6A modifications. The m6A reader protein YTHDC1 can transport mRNAs with m6A methylation modifications from the cell nucleus to the cytoplasm for export [10]. YTHDC2 is another member of the YTH protein family, which selectively binds to m6A modifications within its conserved domains. This binding not only enhances the translation efficiency of its target RNAs but also reduces their overall quantity. Consequently, YTHDC2 regulates both the translation rate and stability of RNA within the cell [11]. YT521-B homology domain family 1 (YTHDF1) is also an m6A-binding protein. Research has demonstrated that YTHDF1, through its binding affinity to m6A modification sites within the 3’-untranslated region (3’-UTR) of mRNA, can recruit members of the eIF3 family of eukaryotic initiation factors. This molecular interaction facilitates direct involvement in cap-independent translation initiation [12]. In addition, the YTH domain family 2 (YTHDF2) regulates RNA degradation by selectively recognizing m6A RNA modification sites, distinguishing m6A-modified RNA, and guiding it to degradation pathways [13].YTHDF3, another cytoplasmic m6A-binding protein in the YTH domain family, has elucidated biological functions. Studies have shown that YTHDF1 can collaborate with YTHDF3 to promote protein synthesis. Additionally, YTHDF3 can influence the degradation of m6A-modified messenger RNA mediated by YTHDF2 [14]. The insulin-like growth factor 2 mRNA-binding proteins (IGF2BPs), comprising IGF2BP1, IGF2BP2, and IGF2BP3, form a key family of RNA-binding proteins. These proteins are essential regulators of cell division and metabolic processes, primarily through their ability to stabilize and control the translation of mRNAs for critical regulatory factors. IGF2BPs are not only pivotal in normal developmental processes but also play significant roles in the progression of various diseases [15]. FTO and ALKBH5, as demethylases targeting m6A modification, have garnered significant attention recently. By modulating m6A levels, they actively participate in various biological processes such as RNA splicing, transport, stability, and metabolism. Exploring the intricate biological roles of FTO and ALKBH5 sheds light on the regulatory mechanisms of m6A modification within cells and holds promise for devising novel therapeutic interventions for associated disorders. Thus, understanding these demethylases’ structure, function, and involvement in disease pathogenesis is paramount for unraveling the complexities of m6A modification regulation.

## The structure of FTO and ALKBH5

**Fig. 2:**
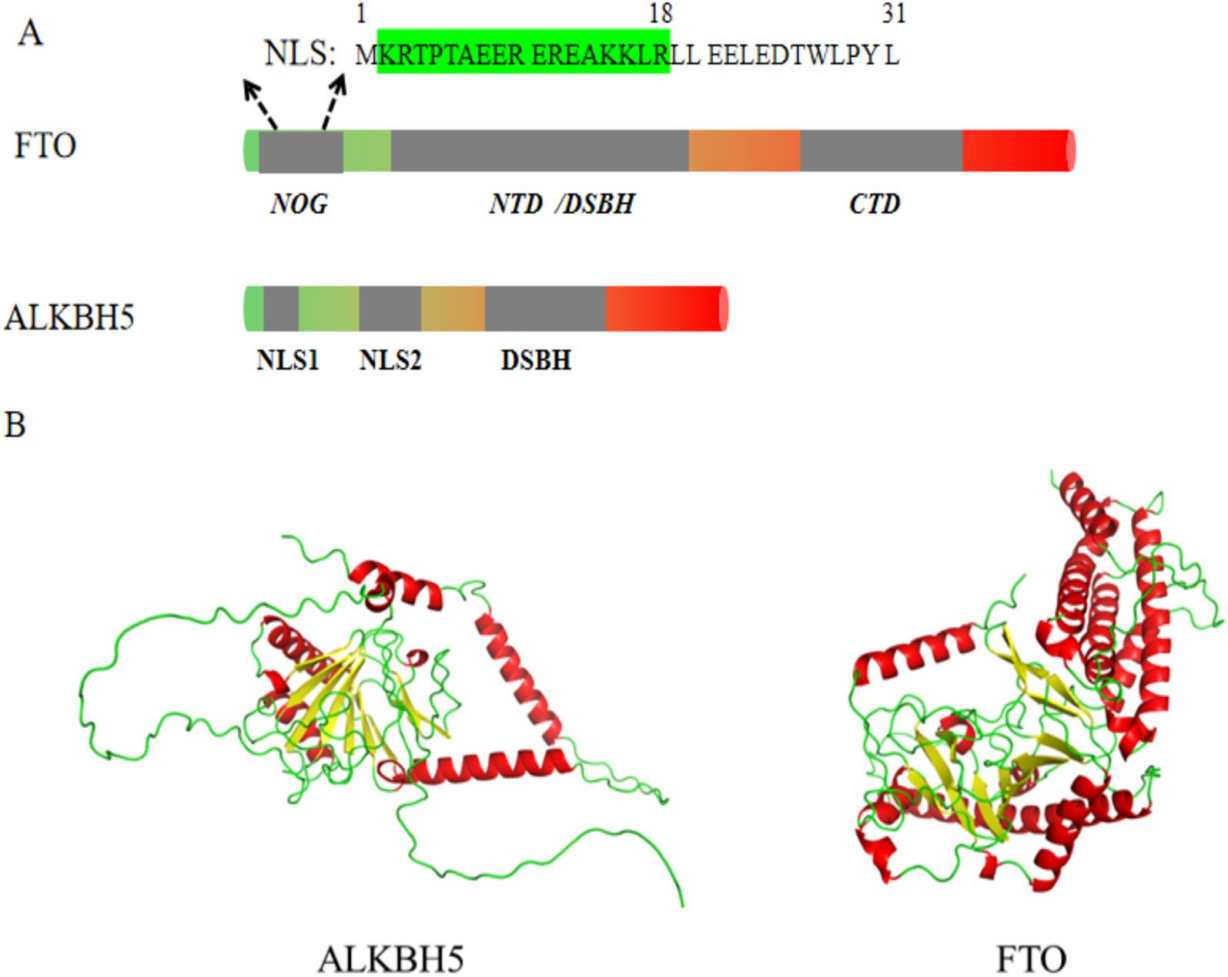
Structures of FTO and ALKBH5. **A** In FTO and ALKBH5, DSBH represents the double-stranded β-helix domain, NOG stands for the N-oxoglutarate binding domain, and CTD represents the carboxyl-terminal domain. The nuclear localization signal is the specific NLS sequence in the NOG structure of FTO. **B** The protein secondary structures of FTO and ALKBH5 predicted by Alphafold are shown here. In the visualization, alpha helices are depicted in red, beta sheets in yellow, and disordered regions in green

FTO and ALKBH5 belong to the AlkB family of Fe(II) and α-ketoglutarate-dependent dioxygenases, capable of removing alkyl adducts from bases through oxidative demethylation [16]. The AlkB family comprises nine AlkB homologs, with the first eight labeled as ALKBH1-8 and the ninth known as FTO (fat mass and obesity-associated protein) [17, 18]. Both FTO and ALKBH5 possess conserved double-stranded β-helix (DSBH) domains, which regulate their demethylase activity (Fig. 2), along with additional functional domains that indirectly impact their demethylation functions [19]. While both enzymes serve as “erasers” of N6-methyladenosine (m6A) modifications, they exhibit significant differences in their secondary structures (Fig. 2).

### The structure of FTO

The crystal structure of the FTO protein was discovered in 2010 [20]. FTO is predominantly distributed in adult and fetal liver, adipose tissue, hypothalamus, pancreas, skeletal muscle, and macrophages [21]. The FTO protein comprises 505 residues and can be mainly divided into three parts: the N-terminal domain (NTD, residues 32–326), the C-terminal domain (CTD, residues 327–984), and the N-oxoglutarate (NOG) domain (residues 1–31) [9]. N-oxoglutarate (NOG) forms the catalytically inert FTO-substrate complex, and the absence of the N-terminal 31 residues does not affect FTO catalytic activity [20]. Demonstrated within the NOG structure, a typical nuclear localization signal (NLS) was found at the N-terminal (residues 2–18). This region is rich in K/R residues and is located within the N-terminal extension, which is conserved from fish to mammals, including humans(Fig. 2) [22]. Experimental evidence indicates that deleting the N-terminal nuclear localization sequence (NLS) results in cytoplasmic localization of FTO [23]. Subsequent studies revealed that Exportin 2 (XPO2) is a binding partner of FTO, mediating protein shuttling between the nucleus and cytoplasm by interacting with the NLS [24]. The subsequent NTD domain contains a conserved double-stranded β-helix (DSBH) structure formed mainly by a twisted arrangement of double-stranded β-helices (β5-β12), also known as the jelly roll motif. Two β-strands (β3 and β4) support this motif on one side [20]. Furthermore, within the NTD, highly preserved residues, including His 231, Asp 233, and His 307, coordinate with Fe21. Beyond Fe21 chelation, NOG forms salt bridges with Arg 316 and Arg 322. Conversely, the C-terminal domain (CTD) predominantly adopts a three-helix bundle configuration comprising α-helices. One extremity of this helical bundle extensively interacts with the NTD, stabilizing its conformation and modulating the enzyme’s activity [20].

### The structure of ALKBH5

The crystal structure of human ALKBH5, comprising 395 amino acids, was elucidated in 2013 [9].ALKBH5 is primarily expressed in the testes and lungs, followed by the spleen, kidneys, and liver, with minimal expression in the heart and brain [21]. FTO and ALKBH5 are both members of the Fe(II) and 2-oxoglutarate (2OG)-dependent dioxygenase family, featuring a double-stranded β-helix (DSBH) domain. However, the 2OG binding site within the DSBH domain differs structurally between FTO and ALKBH5. ALKBH5 with residues 66–292, features a double-stranded β-helix core fold, characteristic of the 2OG and iron-dependent dioxygenase family. The DSBH core fold of ALKBH5, featuring eight antiparallel β-strands (βI-VIII or β6–13), consists of two β-fold structures: primary (β6, 8, 11, and 13) and minor (β7, 9, 10, and 12) β-sheets [25]. The core structure of ALKBH5 also features two nucleotide recognition loops (NRL1 and NRL2), typical of the 2OG dioxygenase AlkB family. Located within ALKBH5’s N-terminal extension, NRL1 is formed by β-strands 2 and 3, while NRL2 is composed of β-strands 4 and 5. Compared to other AlkB subfamily members, ALKBH5’s NRL1 is shorter, and NRL2 is partially disordered [26]. The βIV-V loop of ALKBH5, containing essential residues Lys231, Lys235, and Arg238, contributes to its selectivity for ssRNA [25].ALKBH5’s preference for single-stranded RNA is attributed to a unique “lid region” alongside its β-fold structure, crucial for substrate recognition and catalysis [27]. During ALKBH5 activity, metal ions coordinated by His204, Asp206, and His266, and 2OG forms electrostatic and hydrogen bonds with Asn193, Tyr195, Arg277, and Arg283, stabilizing the structure [28].Sulfate ions are also found to interact with Asn193 and Lys132 [26]. Structural and active site differences may give FTO and ALKBH5 distinct RNA recognition and demethylation specificities. This implies varying affinities and catalytic activities for different RNA substrates. In summary, despite both being m6A demethylases, FTO and ALKBH5 have significant structural differences. Understanding these differences can improve our insight into their regulatory roles in post-transcriptional RNA modifications.

## The demethylation process of m6A by FTO and ALKBH5

**Fig. 3:**
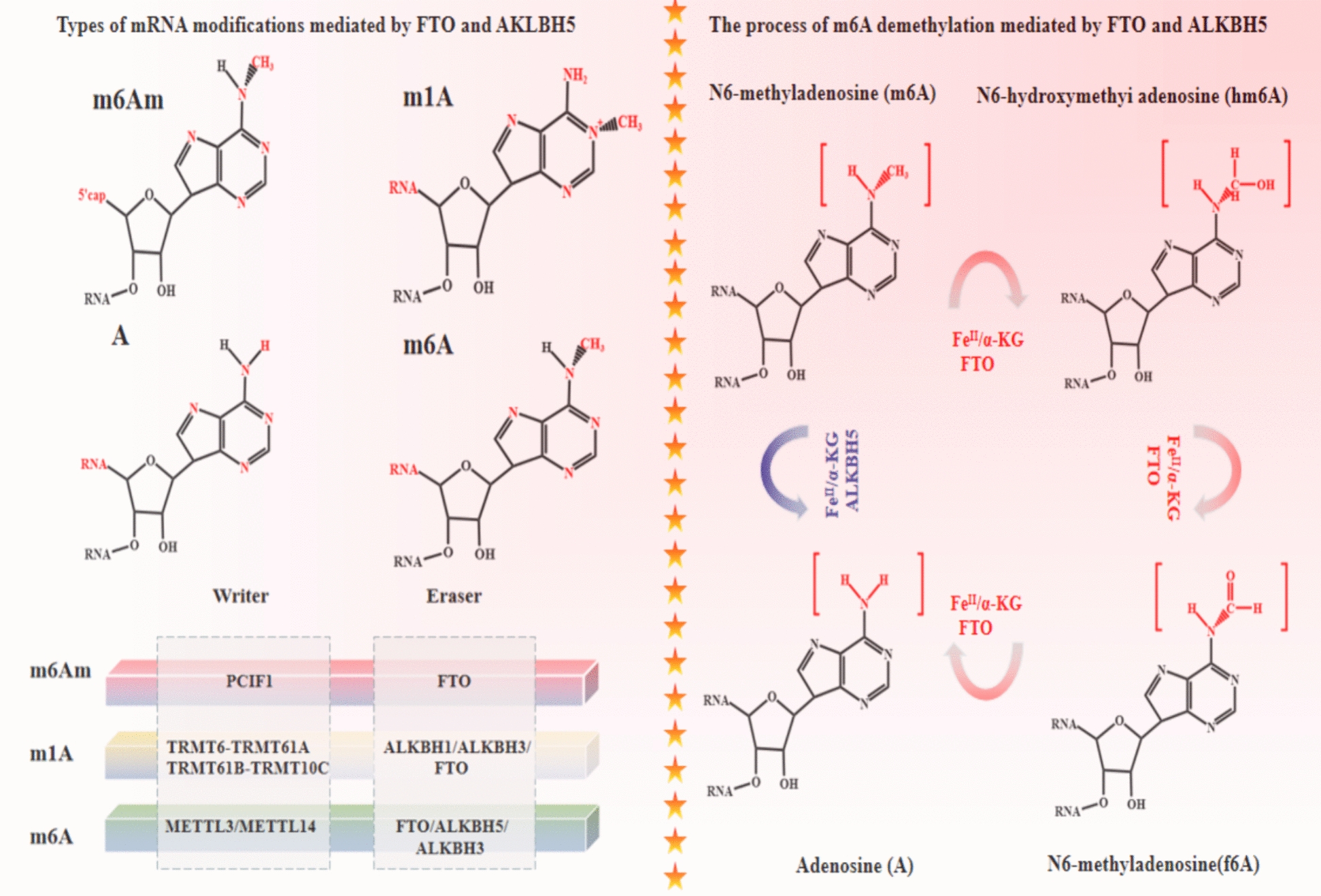
Summarize the types of RNA modifications mediated by FTO and ALKBH5, and elaborate on the different RNA modifications mediated by the two demethylases, emphasizing their recognition of methyl positions on different substrate RNAs. Additionally, discuss the dynamic processes of FTO and ALKBH5 in recognizing M6A methyl groups

In 2011, He et al. identified FTO as the first m6A demethylase, demonstrating its efficient oxidative demethylation activity in vitro on nuclear RNA [29]. Besides its role in m6A methylation, FTO influences snRNA m6A and m6Am levels and mediates tRNA m1A demethylation [30] (Fig. 3). Experimental evidence indicates that FTO primarily targets m6A in nuclear polyadenylated RNA. In the cytoplasm, however, it also demethylates m6A in mRNA, mainly targeting the m6Am cap [31]. Cytoplasmic FTO inhibits cancer stem cell (CSC) capabilities in colorectal cancer via its m6Am demethylase activity [32]. Furthermore, it has been confirmed that the FTO protein possesses a nuclear localization signal (NLS), aiding its shuttling between the nucleus and the cytoplasm, thereby exerting multiple modifying functions [24]. Recent studies reveal that FTO interacts with transcription factor FOXO3a, boosting its activity and reducing glioma invasiveness [33]. These findings suggest that FTO has diverse biological functions, including regulating RNA methylation status, affecting cellular metabolism and stability, and playing significant roles in diseases such as cancer.

In 2012, ALKBH5 was identified as a mammalian RNA demethylase that catalyzes the oxidative reversal of m6A in mRNA, affecting RNA metabolism and mouse fertility, both in vitro and in vivo [34]. ALKBH5 functions primarily as a specialized m6A demethylase. FTO and ALKBH5 follow different pathways in catalyzing RNA oxidation N-demethylation. FTO employs the traditional oxidation N-demethylation pathway, converting m6A to hm6A and slowly releasing A and FA. Conversely, ALKBH5 directly converts m6A to A, quickly releasing FA [35] (Fig. 3). ALKBH5 also demethylates N6,2’-O-dimethyladenosine (m62A) in ribosomal RNA, a noncanonical base [36]. Additionally, ALKBH5 demethylates specific long non-coding RNAs (lncRNAs), such as KCNK15-AS1, which are downregulated in pancreatic cancer, leading to the migration and invasion of cancer cells [37].

## The biological functions of FTO

### FTO and energy metabolism

The 2007 discovery of SNPs in the FTO gene locus, linked to BMI and obesity risk across various populations, marked FTO as the first gene definitively linked to obesity [38]. Subsequent studies revealed that mice with multiple copies of the FTO gene became obese, while deleting this gene prevented obesity [39]. It was later discovered that FTO encodes an m6A RNA demethylase, significantly influencing various biological and metabolic processes [29]. Following its identification as an m6A demethylase, research has uncovered molecular links between FTO’s activity and obesity susceptibility. Studies have shown that FTO modulates early fat formation events by upregulating the pro-adipogenic factor RUNX1T1, thereby promoting preadipocyte proliferation [40].

Additionally, the impact of FTO on glucose metabolism was demonstrated in mice treated with the FTO inhibitor entacapone. These mice exhibited significant weight loss, lower blood sugar levels, and increased thermogenesis. Yang et al. discovered that FTO regulates energy metabolism by controlling G6PC expression through m6A demethylation of FOXO1. Furthermore, FTO inhibitors have been shown to reduce PI3K and Akt levels in breast cancer cells, leading to decreased activities of pyruvate kinase and hexokinase, thereby inhibiting glycolysis [41].

FTO modifies the processing, maturation, and translation of lipid-related gene mRNA. FTO catalyzes the demethylation of m6A, removing its subsequent binding with reader proteins, thereby altering the splicing, maturation, translation, or degradation of substrate mRNA, including lipid-related genes. Overexpressing FTO in HepG2 cells increased fat production by regulating SREBP1c and CIDEC expression, key lipid generation factors [42]. At the same time studies has linked FTO with non-alcoholic fatty liver disease (NAFLD). FTO stabilizes SREBF1 and ChREBP mRNA by demethylating m6A sites, enhancing insulin response and liver fat production. FTO inhibition also improved hepatic steatosis in mice fed a high-fat diet [43].

Additionally, FTO influences lipid cell differentiation by regulating classical signaling pathways. FTO deficiency in porcine and murine preadipocytes inhibits adipogenesis via the JAK2-STAT3-C/EBPβ signaling pathway. Mechanistically, FTO deficiency inhibits JAK2 expression and STAT3 phosphorylation, leading to reduced C/EBPβ transcription, crucial for the early stages of adipocyte differentiation [44]. FTO enhances fat cell proliferation and differentiation in porcine intramuscular preadipocytes by inhibiting the Wnt/β-catenin pathway [45]. Downregulating FTO suppresses mitochondrial biogenesis and energy production by inhibiting the mTOR-pg-1a pathway [46].

### FTO and neurological disorders

FTO plays a crucial role in various biological processes, including brain development. In the nervous system, FTO knockout mice show postnatal growth retardation and decreased brain volume [47]. Evidence links FTO to neuropsychiatric disorders such as Alzheimer’s, Parkinson’s disease, anxiety, depression, and epilepsy. FTO influences Alzheimer’s disease via the TSC1-mTOR-Tau signaling pathway. Conditional Fto knockout in neurons lessened cognitive deficits in 3xTg AD mice [48]. Zan et al. highlighted FTO’s critical role in regulating dopaminergic neuron death in Parkinson’s disease through its m6A demethylase activity. In an ex vivo PD model, FTO levels were elevated in dopaminergic neurons. Knocking down FTO reduced α-Syn expression and neuron apoptosis while enhancing ATM expression via m6A-dependent mRNA stabilization, increasing TH protein levels [49]. Fto deficiency serves as a protective factor against chronic stress in Fto+/- mice, resulting in reduced body weight, decreased anxiety, and depression-like behaviors, highlighting the significance of FTO in depression pathogenesis [50]. In an epilepsy model, Nrf2 and FTO levels were down-regulated. FTO overexpression decreased seizure frequency and hippocampal neuron apoptosis by reducing Nrf2 mRNA m6A methylation [51]. In summary, these findings outline FTO’s specific role in neuropathy, highlighting its potential as a therapeutic target for various neuropsychiatric disorders.

### FTO and cardiovascular diseases

FTO is critically involved in the initiation and progression of cardiovascular diseases, including myocardial fibrosis, heart failure, and atherosclerosis. Increasing evidence suggests a correlation between genetic variations in the FTO gene and cardiovascular disease (CVD) risk. Myocardial fibrosis, characterized by ventricular remodeling, is a primary pathological feature leading to impaired cardiac function. BIE et al. discovered that circCELF1 regulates DKK2 expression via the FTO/m6A and miR-636 pathway, thereby suppressing myocardial fibrosis [52]. Furthermore, reduced FTO expression in heart failure leads to abnormal increases in cardiac m6A levels and m6A in selective contractile transcripts. This highlights FTO’s protective mechanism in the heart, where selective demethylation of myocardial contractile transcripts under ischemic conditions enhances mRNA stability and protein expression, ultimately reducing myocardial fibrosis and promoting angiogenesis [53].

FTO-mediated hypermethylation was demonstrated in an experimental model of DBDPE-induced glucose and lipid metabolic disorders, leading to myocardial dysfunction, cardiomyocyte fibrosis, and apoptosis through a mitochondrial-mediated apoptotic pathway [54]. Moreover, upregulation of FTO significantly reduces cholesterol ester accumulation in macrophages loaded with oxidized low-density lipoprotein and prevents atherosclerotic plaque formation while markedly decreasing plasma levels of total cholesterol and low-density lipoprotein cholesterol, predominantly in male atherosclerotic mice [55]. In HFpEF mice, FTO was found to be upregulated [56]. Furthermore, experimental evidence demonstrated that elevated FTO levels inhibited apoptosis in myocardial cells subjected to hypoxia/reoxygenation. Specifically, FTO overexpression suppressed myocardial cell apoptosis induced by hypoxia/reoxygenation by modulating the m6A modification of Mhrt, thereby ameliorating heart failure [57]. Additionally, FTO mitigates heart dysfunction in pressure-overloaded heart failure mice by regulating glucose uptake and glycolysis [58].

### FTO and cancer

FTO was initially discovered in the early 1990s as the first RNA-m6A demethylase involved in lipid regulation, and it was later found to play a significant role in cancer. Dysregulation of FTO is observed in various cancers, including acute myeloid leukemia (AML), glioblastoma, cervical squamous cell carcinoma (CSCC), gastric cancer, and small cell lung cancer. Changes in FTO expression can occur under disease conditions, affecting downstream target genes and thereby promoting tumor progression and initiation (Table 1).

**Table 1:** Roles of RNA m6A-FTO / ALKBH5 in cancers

The m6A demethylase	Cancers	Role	Molecular mechanism of tumor progression	References
FTO	On-small cell lung cancer	Oncogene	FTO boosts E2F1 expression by inhibiting its m6A modification, enhancing the viability, migration, and invasion of NSCLC cells	[59]
FTO	Esophageal squamous cell carcinoma	Oncogene	FTO overexpression reduces LINC00022 m6A methylation, stabilizing its transcript and promoting cell cycle progression and proliferation	[60]
FTO	Acute Myeloid Leukemia	Oncogene	FTO reduces m6A levels, affecting oncogene-driven transformation and leukemia development	[61]
FTO	Gastric cancer	Oncogene	FTO reduces m6A methylation on MYC mRNA, stabilizing its expression and promoting gastric cancer cell growth and spread	[62]
FTO	Gastric cancer	Oncogene	FTO promotes the degradation of CAV1 mRNA, thereby regulating mitochondrial fission/fusion and metabolism	[63]
FTO	Cervical cancer	Oncogene	FTO regulates m6A modification levels of ZEB1 and Myc, affecting cell proliferation, migration, and invasion in cervical cancer	[64]
FTO	Endometrial cancer	Oncogene	FTO promotes endometrial cancer metastasis by regulating HOXB13 mRNA stability through m6A modification	[65]
FTO	Breast cancer	Antioncogene	FTO reduction leads to increased m6A modification, thereby regulating the expression of the critical gene KRT7 and promoting the metastasis of breast cancer cells to the lungs	[67]
FTO	Colorectal cancer	Antioncogene	colorectal cancer suppresses the metastasis of colorectal cancer by inhibiting the expression of metastasis-associated protein 1 (MTA1)	[68]
FTO	Ovarian Cancer	Antioncogene	FTO decreases m6A levels and regulates the stability of PDE1C and PDE4B genes, thereby enhancing cAMP signaling and suppressing ovarian cancer cell stemness	[69]
ALKBH5	Gastric cancer	Oncogene	ALKBH5 removes m6A modifications from JAK1 mRNA, leading to the upregulation of JAK1 gene expression and promoting the proliferation and metastasis of gastric cancer cells	[75]
ALKBH5	Cervical cancer	Oncogene	ALKBH5 mediates the regulation of m6A modification on CircCDC134, enhancing its stability and further promoting the proliferation and metastasis of cervical cancer	[76]
ALKBH5	AML	Oncogene	Promoter ALKBH5 demethylates ITPA mRNA, increasing its stability and enhancing ITPA expression	[77]
ALKBH5	Pancreatic cancer	Oncogene	The role of ALKBH5 in this process is to demethylate DDIT4-AS1 mRNA and increase its stability, thereby leading to the upregulation of DDIT4-AS1	[78]
ALKBH5	Orchestrates glioma	Oncogene	USP36 regulates the protein degradation and expression of ALKBH5, and the USP36-ALKBH5 axis orchestrates glioma tumorigenesis	[79]
ALKBH5	Pancreatic neuroendocrine neoplasms	Oncogene	ALKBH5 can activate the PI3K/Akt/mTOR signaling pathway, enhancing lipid metabolism and proliferative capacity	[80]
ALKBH5	Non-small cell lung cancers	Oncogene	ALKBH5 mediates the m6A modification of SOCS2, mediating the activation of the interleukin-6-regulated JAK-STAT pathway	[81]
ALKBH5	Ovarian cancer	Oncogene	ALKBH5 increases the expression of ITGB1, thereby enhancing lymph node metastasis in ovarian cancer	[82]
ALKBH5	Breast cancer	Oncogene	ALKBH5's activity increases NANOG mRNA's stability and NANOG protein levels, thereby increasing the number of breast cancer stem cells (BCSCs)	[83]
ALKBH5	Glioblastoma Stem-like Cells	Oncogene	ALKBH5 demethylates nascent transcripts of FOXM1, leading to enhanced FOXM1 expression	[84]
ALKBH5	Pancreatic cancer	Antioncogene	ALKBH5 acts through a m6A-YTHDF2-dependent mechanism to demethylate the PER1 gene post-transcriptionally, leading to upregulation of PER1	[85]
ALKBH5	Non-Small Cell Lung Cancer	Antioncogene	ALKBH5 inhibits tumor growth and metastasis in vivo by reducing the expression and activity of YAP	[86]
ALKBH5	Hepatocellular carcinoma	Antioncogene	ALKBH5-mediated m^6^A demethylation led to a post-transcriptional inhibition of LY6/PLAUR Domain Containing 1 (LYPD1)	[87]
ALKBH5	Human osteosarcoma	Antioncogene	ALKBH5 inactivated the STAT3 pathway by increasing SOCS3 expression in an m6A-YTHDF2-dependent manner	[88]

#### FTO as an oncogene

FTO, a key regulator of m6A modifications, exhibits both oncogenic and tumor-suppressive properties across various cancer types. FTO acts as an oncogene in certain cancers by altering the m6A modification of crucial transcripts, thereby promoting cancer progression. Recent studies underscore FTO’s crucial role in lung cancer. Consequently, FTO increases E2F1 mRNA stability and activity, leading to E2F1 protein overexpression. These actions profoundly influence the biological characteristics of NSCLC cells by enhancing their vitality and promoting their migration and invasion [59]. In esophageal squamous cell carcinoma (ESCC), FTO is highly expressed and associated with the demethylation of LINC00022, thereby promoting tumor growth in vivo [60]. In AML, especially MLL-rearranged subtypes, FTO plays a critical oncogenic role by enhancing AML cell vitality and proliferation while inhibiting apoptosis, contributing to disease development [61]. Elevated FTO expression in GC tissues correlates with increased expression of factors promoting cancer development, such as HDAC3 and MYC, while the transcription factor FOXA2 expression decreases. The primary regulatory mechanism involves FOXA2 binding to the FTO gene promoter, reducing FTO expression. Additionally, FTO stabilizes MYC mRNA by reducing m6A methylation in GC cells, enhancing cell vitality, migration, and invasion [62]. Additionally, FTO’s activity promotes the degradation of vesicle-associated membrane protein-1 mRNA, affecting mitochondrial dynamics and metabolic functions, and contributing to the progression of gastric cancer [63]. FTO’s oncogenic potential extends to cervical and endometrial cancers, where it interacts with transcription factors such as E2F1 and MYC to regulate tumor cell proliferation and migration [64]. In endometrial cancer (EC), higher FTO expression is closely linked to tumor metastasis and invasion. FTO catalyzes the demethylation of the HOXB13 mRNA 3ʹUTR region, affecting m6A recognition by the YTHDF2 protein. This promotes the activation of the WNT signaling pathway, increases downstream protein expression, and enhances tumor metastasis and invasion [65]. FTO also plays a role in regulating tumor cell metabolism, immune evasion, and metastasis. Studies indicate that FTO promotes glycolysis in tumor cells while limiting T-cell immune responses against tumors [66].

#### FTO as an antioncogene

On the other hand, FTO exhibits tumor-suppressive functions in various cancer contexts. In pulmonary metastases of breast cancer cells, it was found that METTL3 expression is increased while FTO expression is decreased. METTL3-mediated m6A modification enhances the stability of KRT7-AS/KRT7 mRNA through the IGF2BP1/HuR complex, whereas FTO regulates the translation elongation of KRT7 mRNA via YTHDF1/eEF-1.Knockdown of METTL3 or overexpression of FTO can inhibit the intra- and extra-tumoral metastasis of breast cancer cells [67]. Similarly, FTO is observed as a tumor suppressor in colorectal cancer. Studies have shown that FTO protein levels are downregulated in colorectal cancer tissues. FTO suppresses the expression of metastasis-associated protein 1 (MTA1) in an m6A-dependent manner, thereby exerting an anti-tumor effect [68].

Additionally, FTO expression is suppressed in ovarian tumors and cancer stem cells (CSCs). FTO inhibits the self-renewal of ovarian CSCs in vivo, suppressing tumor initiation, both of which require the demethylase activity of FTO. FTO enhances the second messenger 3’,5’-cyclic adenosine monophosphate (cAMP) signaling by reducing the m6A levels of the 3’UTR and stabilizing the mRNA of two phosphodiesterase genes (PDE1C and PDE4B), thereby suppressing the stemness of ovarian cancer cells [69].

### FTO and immune-related diseases

Regulation of RNA modifications has emerged as a critical factor in immune-related diseases. FTO, an enzyme responsible for demethylating m6A, plays diverse roles in physiology and pathology, particularly in immune-related conditions. Normal immune function relies on the precise regulation of cytokines, surface receptors, and co-stimulatory/inhibitory molecules for homeostasis. Recent studies have identified RNA modification as a new regulator of CD40 ligand (CD40L) expression in CD4 + lymphocytes. FTO (an m6A “eraser”) and METTL3 (an m6A “writer”) directly affect CD40L expression, while YTHDF2 (an m6A “reader”) promotes its degradation [70]. The IL-17 signaling pathway, which is associated with inflammation, is crucial in diseases such as NASH and ALD-induced HCC. Studies show that FTO overexpression is correlated with increased IL-17RA mRNA demethylation and protein levels in chronic liver inflammation. Conversely, inhibiting FTO reduces IL-17RA expression, indicating FTO’s role in modulating hepatic inflammation [71]. Moreover, miR-495 inhibits FTO in macrophages, promoting M1 polarization and suppressing M2, which worsens insulin resistance and inflammation in T2D mice [72]. FTO deficiency impedes both M1 and M2 macrophage polarization by lowering NF-κB signaling and reducing the expression of critical genes such as STAT1, STAT6, and PPAR-γ. FTO knockout accelerates the degradation of STAT1 and PPAR-γ mRNAs, while YTHDF2 silencing stabilizes these mRNAs. This underscores FTO’s significant role in regulating macrophage polarization, offering new insights for treating inflammatory diseases [73]. The type I interferon (IFN) signaling cascade activates numerous ISGs critical for antiviral responses. However, excessive activation of type I IFN can lead to inflammation and autoimmune diseases. FTO regulates the type I IFN response by removing m6A or adjacent cap m6Am RNA modifications, thereby inhibiting certain ISGs, including pro-inflammatory genes, primarily by downregulating STAT3 activation and inducing specific ISGs [74] .

## The biological functions of ALKBH5

### ALKBH5 and reproductive system diseases

In 2013, He et al. identified ALKBH5 as a critical mammalian m6A RNA demethylase, with significant implications for mRNA export, fertility, and the prevention of aberrant RNA splicing. ALKBH5 deficiency in male mice leads to increased m6A mRNA levels, impairing meiotic metaphase spermatocyte apoptosis and reducing fertility [34]. This discovery revealed the protective role of ALKBH5-mediated m6A removal, preventing aberrant splicing in longer transcripts within spermatocyte and round spermatid nuclei [89]. Further research demonstrated that the absence of ALKBH5 causes extensive meiotic defects in oocytes, resulting in female infertility. Increased m6A levels following ALKBH5 knockdown, along with the m6A reader IGF2BP2 stabilizing specific transcripts with persistent m6A modifications in oocytes, were linked to this condition [90].

Additionally, studies on recurrent miscarriage (RM) patients revealed a significant decrease in placental villous tissue mRNA m6A methylation levels without specific ALKBH5 expression regulation. Interestingly, ALKBH5 downregulation enhanced trophoblast invasion, while overexpression countered this, showing the RNA regulatory mechanism’s dependence on m6A modifications. Further research shows that in nourish cells reduce ALKBH5 can extend CYR61 mRNA half-lives, increase its expression [91]. ALKBH5 expression was found to increase in trophoblasts under hypoxia but decrease in EVT of patients with recurrent spontaneous abortion (RSA). This suggests ALKBH5 enhances trophoblast activity and reduces spontaneous abortion risk by increasing SMAD1/5 expression via m6A modification removal [92].

### ALKBH5 and central nervous system

ALKBH5 plays a crucial role in the central nervous system (CNS). It is widely distributed in mouse brains, primarily in neurons, and alongside the NeuN neuronal marker. ALKBH5 protein levels significantly decrease during brain development, indicating its importance in CNS functions [93]. ALKBH5 is active in synaptic ribosomes during plasticity, participating in the synaptic labeling hypothesis by interacting more with YTHDF1 and YTHDF3 [94]. The intrinsic regenerative capacity of the PNS and CNS is a crucial factor limiting axonal regeneration success. In the PNS, ALKBH5 knockdown enhanced sensory axon regeneration, while its overexpression impaired it in an m6A-dependent manner. In the CNS, ALKBH5 down-regulation promoted retinal ganglion cell survival and axon regeneration post-optic nerve injury [95]. Rising RNA m6A levels correlated with Alkbh5 expression in rat brain tissues under arterial occlusion or hypoxia/reoxygenation. Knockdown of ALKBH5 increased neuronal damage levels, the study showed. This results from ALKBH5 and FTO demethylation, selectively degrading and stabilizing Bcl2 transcripts, thus increasing Bcl2 protein levels [96]. This further confirms the vital role of ALKBH5 in developing neural prominence. Recent studies indicate ALKBH5 regulates trigeminal nerve-mediated neuropathic pain functions. Nerve injury down-regulates histone deacetylase 11, increasing H3K27ac acetylation and promoting FOXD3 binding to the ALKBH5 promoter, enhancing ALKBH5 transcription. Increased ALKBH5 erases m6A sites in Htr3a mRNA, preventing YTHDF2 binding, thus raising 5-HT3A protein expression and channel currents. FOXD3 activates ALKBH5 in TG neurons via m6A-dependent Htr3a mRNA stabilization, promoting neuropathic pain [97].

### ALKBH5 as an oncogene

ALKBH5, known for its role in reversing m6A modifications in mRNA, significantly impacts mRNA export, RNA metabolism, and the assembly of mRNA processing factors in nuclear speckles [34]. Studies indicate ALKBH5’s abnormal expression in various cancers, including gastric cancer, t(8;21)AML, pancreatic neuroendocrine tumors, and glioblastoma, linking it to tumorigenesis and progression (Table 1). In gastric cancer, high ALKBH5 expression correlates with aggressive clinical features and poor prognosis. Both ex vivo and in vivo studies confirmed ALKBH5’s role in promoting gastric cancer cell proliferation and metastasis. Specifically, ALKBH5 upregulated JAK1 expression by demethylating JAK1 mRNA. LINC00659 facilitated ALKBH5’s binding and upregulation of JAK1 mRNA in an m6A-YTHDF2-dependent manner. Silencing ALKBH5 or LINC00659 disrupted the JAK1 axis, inhibiting gastric cancer tumorigenesis and progression [75]. In cervical cancer, high ALKBH5 expression is linked to aggressive features and poor prognosis. ALKBH5-mediated demethylation destabilizes circCCDC134 via YTHDF2, significantly impacting cervical cancer metastasis [76]. ALKBH5 also shows high expression in t(8;21) AML patients. Silencing ALKBH5 inhibited proliferation and promoted apoptosis in patient-derived AML and Kasumi-1 cells. Integrative transcriptome analysis identified ITPA as a key functional target of ALKBH5. ALKBH5 demethylated and stabilized ITPA mRNA, enhancing ITPA expression. Additionally, TCF15, expressed in leukemia stem cells, was linked to aberrant ALKBH5 regulation in t(8;21) AML [77]. In pancreatic ductal adenocarcinoma (PDAC), ALKBH5 regulates the development and treatment response by modulating DDIT4-AS1. High ALKBH5 expression correlates with PDAC malignancy and poor prognosis. Silencing DDIT4-AS1 reduced PDAC cell stemness and increased sensitivity to gemcitabine. ALKBH5’s demethylation activity stabilizes DDIT4-AS1, influencing PDAC cell behavior [78]. Aberrant activation of ALKBH5 in glioblastoma (GBM) plays a critical role in tumor growth and progression, and studies have identified a mechanism of regulation of ALKBH5 by USP36. This protease influences the activity of ALKBH5 in GBM by regulating its protein degradation and expression levels [79]. In glioblastoma (GBM), aberrant activation of ALKBH5 plays a critical role in tumor growth and progression. USP36 regulates ALKBH5 activity in GBM by controlling its protein degradation and expression levels. ALKBH5 also exhibits upregulated expression in pancreatic neuroendocrine tumors (pNENs), playing a critical role in tumor growth and lipid metabolism. ALKBH5 overexpression increases FABP5 expression in an m6A-IGF2BP2-dependent manner, disrupting lipid metabolism. It also activates the PI3K/Akt/mTOR signaling pathway, enhancing lipid metabolism and proliferation [80]. Extracellular vesicles (EVs) released from cigarette smoke extract (CSE)-induced M2 macrophages affect ALKBH5 activity in non-small cell lung cancer (NSCLC). CircEML4 carried in these EVs interacts with ALKBH5, reducing its nuclear distribution and increasing m6A modification levels. This activates the Janus kinase signaling pathway by regulating the SOCS2 gene, contributing to malignant metastasis and tumor growth in NSCLC [81]. In ovarian cancers with lymph node metastasis, m6A modification levels decrease. Overexpression of ALKBH5 significantly increases tumor-associated lymphangiogenesis and lymph node metastasis in vitro and in vivo. ALKBH5 reverses m6A modification in ITGB1 mRNA, inhibiting YTHDF2-mediated degradation and promoting ITGB1 expression. This enhances the phosphorylation of adhesion kinase (FAK) and Src proto-oncogene proteins, increasing lymph node metastasis. Hypoxia induces hypoxia-inducible factor 1 subunit expression, which increases ALKBH5 levels in ovarian cancer cells, further enhancing lymph node metastasis [82]. Hypoxia-inducible factor (HIF)-1α and HIF-2α upregulate ALKBH5 expression in breast cancer cells under hypoxic conditions. As an m6A demethylase, ALKBH5 predominantly demethylates NANOG mRNA at the 3’-UTR, encoding pluripotency factors. Hypoxia increases NANOG mRNA and protein expression through a HIF- and ALKBH5-dependent mechanism, increasing the proportion of breast cancer stem cell (BCSC) phenotypes [83]. In glioblastoma stem-like cells (GSCs), high expression of ALKBH5 is closely related to malignant characteristics. Its deletion significantly inhibited GSC proliferation. ALKBH5 regulates the expression of important genes, including the transcription factor FOXM1. ALKBH5 removes the m6A modification of FOXM1 nascent transcripts and enhances FOXM1 expression. In addition, long chain antisense noncoding RNA (FOXM1 - AS) can also by promoting ALKBH5 interact with FOXM1 transcription of this new further regulate the expression of FOXM1 [84].

### ALKBH5 as an antioncogene

As a tumor suppressor gene, the expression level of ALKBH5 is closely associated with tumor progression, patient prognosis, and therapeutic outcomes. Studies have shown that in various types of cancer, low ALKBH5 expression is often linked to tumor deterioration and poor prognosis, while high ALKBH5 expression can significantly inhibit tumor cell proliferation, migration, and invasion, thereby slowing down tumor growth. In pancreatic cancer, low ALKBH5 levels are associated with adverse clinical outcomes, while its overexpression inhibits tumor cell proliferation, migration, invasion, and tumor growth. Mechanistically, ALKBH5 activates the PER1 gene through m6A-YTHDF2-dependent post-transcriptional modification. This upregulation reactivates the ATM-CHK2-P53/CDC25C signaling cascade, hindering tumor proliferation. Additionally, p53-induced ALKBH5 transcription forms a feedback loop, modulating m6A levels and affecting tumor development [85].In NSCLC, ALKBH5 expression inversely correlates with AP expression, indicating its tumor-suppressive role. Specifically, ALKBH5 reduces the m6A modification level of YAP. ALKBH5 attenuates YAP activity by regulating the miR-107/LATS2 axis through a HURR-dependent mechanism. This regulatory mechanism inhibits YAP-mediated tumor cell proliferation, invasion, migration, and epithelial-mesenchymal transition (EMT). Moreover, ALKBH5 curbs in vivo tumor growth and metastasis by reducing YAP expression and activity [86]. In HCC, decreased ALKBH5 expression correlates with poorer survival rates, becoming an independent prognostic factor. Functionally, ALKBH5 suppresses HCC cell proliferation and invasion. Mechanistically, ALKBH5 suppresses LYPD1 post-transcriptionally through m6A demethylation, with stabilization by IGF2BP1. Notably, LYPD1 expression promotes carcinogenic behavior more than ALKBH5 [87]. Low ALKBH5 expression is linked to poorer survival in osteosarcoma patients. Elevating ALKBH5 levels in osteosarcoma cells reduces m6A mRNA levels, inhibiting proliferation, inducing apoptosis, and causing cell cycle arrest. Further research identified SOCS3 as a downstream target of ALKBH5, negatively regulating STAT3 through m6A modification. YTHDF2 recognizes m6A-modified SOCS3 mRNA, facilitating its decay. ALKBH5 enhances SOCS3 expression and deactivates the STAT3 pathway in an m6A-YTHDF2-dependent manner [88].

### ALKBH5 and immune responses

ALKBH5 significantly regulates immune responses. Studies show ALKBH5 modifies interferon-γ and CXCL2 mRNA m6A, which is crucial for CD4^+^ T cell pathogenicity and neutrophil CNS entry. ALKBH5 loss increases these mRNAs’ m6A, reducing stability and protein levels in CD4 + T cells, weakening their response and neutrophil migration. This highlights ALKBH5’s unique role in modulating CD4^+^T cell pathogenicity in autoimmune processes [98]. Additionally, ALKBH5 depletion promotes γδ T cell proliferation, enhancing Salmonella typhimurium infection protection. T Findings suggest ALKBH5 deficiency promotes γδ T cell precursor development and proliferation by impairing Jagged1/Notch2 signaling, expanding mature γδ T cell pools [99]. Furthermore, ALKBH5 critically enhances neutrophil migration against microbial defense. ALKBH5 deficiency increases mortality in CLP-induced sepsis mice, showing higher bacterial loads and cytokines due to reduced neutrophil migration. ALKBH5-deficient neutrophils show reduced CXCR2 expression, impairing migration towards CXCL2 [100].

### Distinguishing features of FTO and ALKBH5 in RNA demethylation

FTO and ALKBH5 are two distinct proteins involved in the demethylation modification of RNA, exhibiting several notable differences: FTO and ALKBH5 belong to the AlkB family of Fe(II)- and ketoglutarate-dependent dioxygenases. However, their structural variances, including differences in amino acid sequences, may influence their selective specificity for the coding sequence. FTO is mainly localized in the nucleus and cytoplasm, whereas ALKBH5 is mainly distributed in the nucleus. FTO and ALKBH5 have different specificities for substrate RNAs; FTO can mediate m6A of mRNA, m6 A and m6 Am of snRNA, and m1 A of tRNA [30], whereas ALKBH5 mainly catalyzes the demethylation of mRNAs during the demethylation process. Meanwhile, ALKBH5 recognizes the coding sequence with a particular pattern, and ALKBH5 binds preferentially to the distal fifth end of the coding sequence [101]. Qian et al. found that m6a demethylation requires RBM33, a previously unrecognized m6 A binding protein. The binding protein recognizes m6 A-tagged RNA substrates through its RRM and recruits ALKBH5 for transcript-specific demethylation. It was also found that RBM33 forms a complex with ALKBH5 and inhibits ALKBH5-SUMOylation and activates ALKBH5-demethylase activity by recruiting the desUMOylase SENP1, which further fills in the ALKBH5 regulatory selection mechanism [102]. Although both are involved in RNA demethylation, FTO and ALKBH5 have different biological roles. FTO predominantly influences energy metabolism and regulation of body weight, whereas ALKBH5 is closely associated with reproductive system functions, tumorigenesis, and development. Interestingly, FTO and ALKBH5 may act as oncogenes or tumor suppressors in different types of cancer, indicating their diverse roles in tumor progression and metastasis. Understanding the distinct mechanisms of action of FTO and ALKBH5 in tumors is crucial for unraveling the molecular underpinnings of cancer development and providing insights for developing targeted therapeutic strategies.

## Regulation of FTO and ALKBH5 expression

### Regulation of FTO expression

FTO is a critical regulator of RNA methylation, and its expression levels are intricately controlled by various functional molecules essential for cellular biological processes. The regulation of FTO affects the level of m6A modification and broadly impacts processes such as cell growth, differentiation, metabolism, and signal transduction (Table 2). First, transcription factors and transcriptional regulators directly affect FTO expression. The zinc finger protein Zfp217 regulates m6A mRNA methylation by activating the transcription of m6A demethylase FTO. Meanwhile Zfp217 loss damage the adipocyte differentiation in 3T3L1 cells [ [103]. In tumor samples of head and neck squamous cell carcinoma (HNSC), it was found that the fat oxidation enzyme FTO is overexpressed and positively regulates the expression of the HOXD1 gene in an m6A-dependent manner. Meanwhile, HOXD1 activates the transcription of the oncogenic factor FTO by directly acting on its promoter region [104]. Furthermore, it has been discovered that the transcription factor FOXA2 can bind to the promoter region of the RNA demethylase FTO gene, leading to a decrease in FTO expression. The regulation of histone deacetylase HDAC3 mediates this downregulation of FTO expression. The activity of HDAC3 maintains the standard transmission of the FTO/m6A/MYC signaling pathway, thereby influencing the development of gastric cancer [62]. A highly conserved binding site for C/EBP has been identified near the transcription start site of the human FTO gene. Chromatin immunoprecipitation (ChIP) experiments have demonstrated that C/EBP can directly bind to the presumed FTO promoter binding region. This suggests that C/EBP may act as a positive regulatory factor, binding to the FTO promoter to enhance gene transcription [105]. The proteasome system also plays a role in regulating FTO expression. Specifically, FTO undergoes post-translational ubiquitination at the Lys-216 position. Introducing a ubiquitin-deficient K216R mutation at this site slows down FTO turnover in HeLa cells. This leads to increased FTO protein levels and decreased nuclear FTO levels, ultimately abolishing FTO nuclear translocation. These findings suggest that the proteasome system regulates FTO stability and intracellular localization by controlling FTO ubiquitination, thereby influencing its function and expression levels within the cell [106].In addition, members of cellular signaling pathways such as the Wnt signaling pathway and factors like STAT3 are also involved in regulating FTO expression. The Wnt signal can induce the formation of a protein complex between EZH2 and β-catenin, which interacts with the LEF/TCF binding elements in the FTO promoter region, thereby inhibiting FTO expression. Downregulation of FTO expression significantly enhances m6A mRNA levels, particularly in metabolic pathway genes such as MYC, ultimately promoting the translation of MYC mRNA [107]. Transcription factor STAT3 is involved in the regulation of FTO expression. STAT3 binds to the FTO promoter, affecting FTO expression levels, thereby regulating m6A modification levels and the chemosensitivity of tumor cells [108].Interestingly, FTO also exerts a regulatory effect on STAT3, displaying opposing effects depending on the specific cellular context and external environmental conditions. On the one hand, in specific scenarios, FTO may act as a negative regulator by inhibiting STAT3-mediated signaling pathways, thereby suppressing cellular inflammation responses and the expression of pro-inflammatory genes, which aids in controlling the inflammatory process [74].On the other hand, in alternative environments, FTO may enhance the stability of STAT3 mRNA, promoting the activation of STAT3-mediated signaling pathways and thereby facilitating cell proliferation and migration, a phenomenon that may hold significance in specific cancer types [109].FTO has been confirmed to exhibit a self-regulatory mechanism. Functioning as a transcriptional repressor, FTO modulates its expression levels by binding to its gene promoter. This binding suppresses gene transcription, effectively inhibiting FTO expression. Notably, this binding is regulated by Fe^2 + rather than 2-OG. In the presence of Fe^2+, it prevents FTO from binding to its promoter, thereby alleviating the transcriptional repression of its gene and leading to increased FTO expression levels [110].

**Table 2:** Regulation of FTO, ALKBH5 expression

RegulatorRegulator	Targets	Expression regulation mechanisms	Disease	References
Zinc finger protein Zfp217	FTO	Zfp217 regulates m6A mRNA methylation by promoting the transcription of the m6A demethylase FTO	Adipogenic differentiation	[103]
HOXD1	FTO	HOXD1 promotes the transcription of the oncogenic factor FTO by directly binding to its promoter region	Head and neck cancer	[104]
FOXA2	FTO	The transcription factor FOXA2 was identified to bind to the FTO gene promoter, decreasing its expression	Gastric cancer	[105]
C/EBPα	FTO	Direct binding of C/EBPα to the putative binding regions in the FTO promoter activates gene transcription		[106].
ubiquitination	FTO	FTO undergoes post-translational ubiquitination on Lys-216, leading to an increase in its level. The FTO K216R mutation resulted in reduced levels of nuclear FTO		[107]
Wnt/β-catenin	FTO	The Wnt signaling pathway triggered the recruitment of β-catenin/TCF/LEF to the TBEs in the FTO promoter region, consequently inhibiting FTO expression	Lung adenocarcinoma	[108].
STAT3	FTO	The binding of STAT3 to the promoter region of FTO facilitates the transcriptional activation of FTO	Breast cancer	[109]
Fe^2 +	FTO	FTO binds to its gene promoter, and this binding is hindered by Fe2 + , leading to increased FTO expression		[112].
HIF-1α、HIF-2α	ALKBH5	Under hypoxic conditions, HIF-1α and HIF-2α regulate the transcription levels of ALKBH5 by directly binding to its promoter region	Breast cancer	[83]
eIF3d	ALKBH5	integrated stress response (ISR) upregulates the expression of ALKBH5 through a translation mechanism directed by eIF3d	Integrated stress response (ISR)	[113]
P53	ALKBH5	P53-induced transcriptional activation of ALKBH5 regulates the expression levels of ALKBH5	Pancreatic cancer	[85]
HBx	ALKBH5	HBV up-regulates ALKBH5 via the HBx-WDR5-H3K4me3 axis	Hepatitis B Virus and liver carcinogenesis	[114]
FOXO3D	ALKBH5	The transcription factor fork head box protein D3 (FOXD3) binds to the ALKBH5 promoter, thereby promoting ALKBH5 transcription	Neuropathic pain	[97]
LKB1	ALKBH5	LKB1 deficiency resulted in the upregulation of ALKBH5 expression through DNA hypermethylation of the CTCF-binding motif located on the ALKBH5 promoter	Lung cancer	[115]
USF1	ALKBH5	USF1 binds to the promoter of ALKBH5, facilitating its transcription activation	Prostate adenocarcinoma	[116]
H3K9me3	ALKBH5	KDM4A binding to the ALKBH5 promoter promotes the transcription of ALKBH5	Intervertebral disc degeneration	[117]

### Regulation of ALKBH5 expression

The regulation of ALKBH5 expression plays a crucial role in various biological processes and diseases (Table 2). Its expression is influenced by environmental conditions such as hypoxia and intracellular and extracellular stress, as well as specific transcription factors. The activity and expression of ALKBH5 are regulated through multiple levels, including post-transcriptional modifications, protein interactions, and non-coding RNAs. These complex networks ensure precise intracellular regulation of ALKBH5, allowing it to adapt to environmental changes and adjust its activity in a timely manner. HIF-dependent ALKBH5 expression mediates the enrichment of breast cancer stem cells (BCSCs) in the hypoxic tumor microenvironment [83]. Additionally, cells respond to internal and external stresses by reducing total protein synthesis and activating genetic programs related to survival. The atypical cap-binding protein eIF3d drives the integrative stress response (ISR). Under prolonged stress, eIF3d upregulates the m6A demethylase ALKBH5 to facilitate 5’UTR-specific demethylation of stress-responsive genes, including ATF4 [111]. Additionally, P53 serves as a critical regulatory switch for ALKBH5 expression. Studies have demonstrated that ALKBH5 deficiency is associated with disease progression and poor clinical outcomes in prostate cancer (PC) patients. Conversely, overexpression of ALKBH5 reduces tumor cell proliferation and invasion while promoting enhanced tumor growth. Molecularly, P53-induced ALKBH5 transcription may facilitate m6A demethylation of PER1 in an m6A-YTHDF2-dependent manner, consequently reactivating the ATM-CHK2-P53/CDC25C signaling pathway and impeding cell growth [85]. Furthermore, ALKBH5 expression has been linked to other diseases, such as hepatitis B-associated hepatocellular carcinoma (HBV-HCC).In this case, the high expression of ALKBH5 is mediated by the hepatitis B virus (HBV) x protein (HBx) and relies on the wdr5-triggered H3K4me3 modification of the ALKBH5 gene promoter. Increased ALKBH5 protein catalyzes m6A demethylation of HBx mRNA, stabilizing and promoting higher levels of HBx expression [112]. The m6A demethylase ALKBH5 was recently discovered to be crucial in regulating trigeminal nerve-mediated neuropathic pain. Upregulation of ALKBH5 in neurons fully induces pain-related behaviors. Mechanistically, nerve injury induces downregulation of histone deacetylase 11, resulting in increased acetylation of H3K27ac, which enhances binding of the transcription factor fork head box protein D3 (FOXD3) to the ALKBH5 promoter and thereby boosts ALKBH5 transcription. Increased ALKBH5 erases the m6A site in Htr3a mRNA, preventing YTHDF2 from binding to Htr3a mRNA, consequently increasing the expression of 5-HT3A proteins and 5-HT3 channel currents [97]. KRAS mutations combined with deletion of the LKB1 tumor suppressor gene (KL) are strongly linked to aggressive forms of lung cancer. In lung tumors, m6A modifications in mRNA play a significant regulatory role, although the mechanism remains incompletely understood. Studies have demonstrated that reduced m6A levels correlate with poor disease progression and survival in KL patients. This correlation is partially mediated by specific increases in the levels of ALKBH5 and certain m6A demethylases. Enhancing or losing ALKBH5 function effectively reverses the regulation of proliferation, colony formation, and migration of KRAS-mutant lung cancer cells by LKB1. The specific mechanism suggests that LKB1 deletion leads to high methylation of the CTCF-binding motif on the ALKBH5 promoter, inhibiting CTCF binding while enhancing histone modifications, including H3K4me3, H3K9ac, and H3K27ac. Restoration of LKB1 expression can effectively reverse these effects [113]. USF1, a widely expressed transcription factor, plays a crucial role in PRAD. Upregulation of USF1 suppressed the glycolytic activity of PRAD cells and decreased cell proliferation and metastasis. Additionally, USF1 enhanced the transcriptional activity of ALKBH5, influencing the m6A demethylation process and regulating the biological behavior of PRAD cells [114]. Intervertebral disc degeneration is a significant contributor to low back pain and disability. Yang et al. identified and demonstrated that upregulation of ALKBH5 induces cellular aging. ChIP -qPCR and DNA-Pulldown techniques revealed that KDM4A-mediated H3K9me3 regulated upregulation of ALKBH5, and inhibition of ALKBH5 expression suppressed cellular aging and halted the degenerative process of the lumbar disc [115] .

## The application prospects of FTO and ALKBH5 inhibitors

### The development of FTO inhibitors

**Fig. 4:**
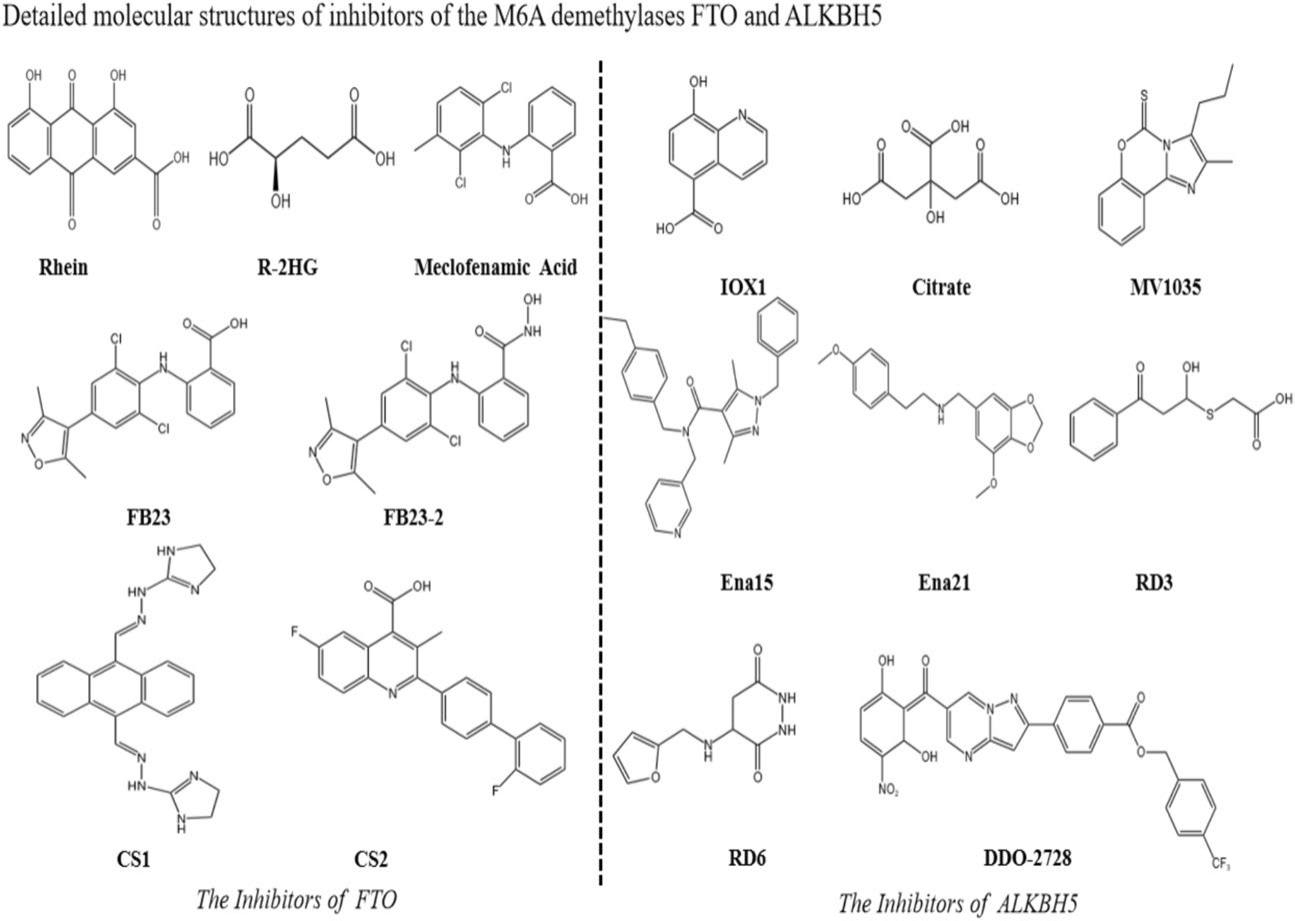
The molecular structure of the FTO and ALKBH5 inhibitors. Significant progress has been made in the research and development of FTO and ALKBH5 inhibitors. Since the discovery of FTO inhibitors in 2012, several inhibitors with potential therapeutic value have been developed, including the natural product Rhein, the metabolite R-2HG, the non-steroidal anti-inflammatory drug Meclofenamic acid (MA), and new inhibitors FB23, FB23-2, CS1, and CS2. Additionally, a glutathione (GSH) bioimprinted nanocomposite material called GNPIPP12MA has shown potential in enhancing anti-leukemia effects.The discovery of ALKBH5 inhibitors has also undergone several important stages. Initial candidate compounds, such as citrate, were identified through high-throughput screening and computer-aided drug design, showing moderate inhibitory effects. Broad-spectrum inhibitor IOX1, sodium channel blocker MV1035, and newly discovered small-molecule inhibitors Ena15, Ena21, RD3, RD6, and DO-2728a have demonstrated significant anti-tumor potential by promoting apoptosis and inhibiting the proliferation of tumor cells

Given the crucial role of FTO in regulating RNA demethylation, its inhibitors have been the focus of extensive research. Since their discovery in 2012, research on FTO inhibitors has shown promising clinical potential. Rhein, a natural product, is a significant FTO inhibitor. Unlike 2-oxoglutarate analogs or metal ion chelators, Rhein inhibits FTO’s m6A demethylation activity by competing with its active site, significantly inhibiting m6A demethylation within cells [116]. Another notable metabolite, R-2HG, inhibits FTO activity under specific conditions, leading to an increase in RNA’s m6A modifications [117]. R-2HG inhibits aerobic glycolysis in sensitive leukemia cells without affecting standard hematopoietic stem and progenitor cells [118]. In subsequent studies, meclofenamic acid (MA) has been identified as a highly selective FTO inhibitor. As an NSAID, meclofenamic acid competitively inhibits FTO-bound nucleic acids’ m6A demethylation process. Treating HeLa cells with mefenamic acid’s ethyl ester (MA2) increased mRNA’s m6A modification levels [119]. In 2019, two potential FTO inhibitors, FB23 and FB23-2, were developed. These inhibitors directly bind to FTO, selectively inhibiting its m6A demethylase activity. In vitro, simulating FTO deficiency, FB23-2 significantly inhibited human AML cell proliferation and promoted differentiation and apoptosis. In xenograft mouse models, FB23-2 significantly inhibited the progression of human AML cells [120]. Two potent small-molecule FTO inhibitors (CS1, CS2) were reported the following year, showcasing robust anti-tumor effects across various cancer types. These inhibitors exhibited sustained and potent inhibition of AML cell viability and FTO demethylase activity. FTO inhibition rendered leukemia cells more sensitive to T cell cytotoxicity and helped overcome immune evasion induced by low methylation agents [121]. Despite progress, the clinical potential of small-molecule FTO inhibitors is limited by moderate activity, toxicity, and low specificity for leukemia stem cells (LSCs). Researchers then developed a glutathione (GSH) bioimprinted nanocomposite material, GNPIPP12MA, loaded with FTO inhibitors. GNPIPP12MA targets the FTO/m6A pathway, synergistically enhancing anti-leukemia effects by depleting GSH. GNPIPP12MA selectively targets LSCs, inducing hypochromic anemia by disrupting cellular redox status. GNPIPP12MA also increases global m6A RNA modification and decreases LSCs’ transcription levels. GNPIPP12MA enhances anti-leukemia immunity and PD-L1 blockade efficacy by promoting cytotoxic T-cell infiltration [122]. The FTO inhibitors discussed, such as Rhein, Meclofenamic acid, FB23, FB23-2, CS1, CS2, and the GSH bioimprinted nanocomposite (GNPIPP12MA), show promise in therapeutic efficacy and clinical applications for inhibiting FTO activity (Fig. 4). Each inhibitor has unique characteristics and action mechanisms, showing potential in treating diseases like cancer. Research on these inhibitors lays a crucial foundation for developing FTO-targeted anti-cancer therapies. Overall, FTO inhibitors are a promising new class of cancer therapeutics, requiring further research and clinical validation for their full potential.

### The development of ALKBH5 inhibitors

The discovery and development of ALKBH5 inhibitors has gone through several important stages. Initially, initial candidate compounds were identified by high-throughput screening and computer-aided drug design. As research progressed, these candidate compounds were optimized to exhibit higher selectivity and inhibitory activity. For example, several promising ALKBH5 inhibitors with potential therapeutic value have been identified in recent years (Fig. 4). Initially, Xu and colleagues identified citrate as a moderately effective ALKBH5 inhibitor. In vitro experiments showed significant activity, with an IC50 value of 488 mmol/L. Subsequently, co-crystal structure analysis revealed that citrate binding to ALKBH5 effectively displaced metal ions and 2OG (2-oxoglutarate), impacting the enzyme’s activity [27]. IOX1 has been confirmed as a broad-spectrum 2-OG dioxygenase inhibitor designed to target crucial enzymes involved in various biological processes. Specifically, IOX1 competes with the cofactor 2-OG, affecting the activity of ALKBH5. This mechanism enables IOX1 to regulate the m6A demethylation process mediated by ALKBH5, playing a critical role in transcriptional regulation and signaling pathways in epigenetics [123]. Subsequently, it was demonstrated that the ALKBH5 inhibitor IOX1 protects against ischemia/reperfusion-induced acute kidney injury (AKI). This suggests that inhibiting ALKBH5 could be a potential strategy for treating AKI [124]. Furthermore, MV1035, a sodium channel blocker, has been confirmed as a novel ALKBH5 inhibitor, exhibiting significant activity in inhibiting ALKBH5. MV1035 significantly reduces the migration and invasion of U87 glioblastoma cell lines [125]. Additionally, through high-throughput screening of pure small-molecule compounds, two new ALKBH5 inhibitors, Ena15 and Ena21, have been identified. These compounds exhibit either non-competitive or competitive inhibition towards 2-oxoglutarate (2OG). Ena21 exhibits minimal inhibitory activity against FTO, while Ena15 enhances FTO’s demethylase activity. Based on the predicted binding sites from the crystal structure of ALKBH5, these two compounds interact with the catalytic site of 2OG in enzyme kinetics. Moreover, knocking down ALKBH5 or using Ena15 or Ena21 to inhibit cell proliferation in glioblastoma-derived cell lines results in decreased cell populations in the synthetic phase of the cell cycle, increased m6A RNA levels, and stabilization of FOXM1 mRNA [126].The significance of compounds 2-(2-hydroxyethylsulfanyl)acetic acid (RD3) and 4-[(methyl)amino]-3,6-dioxo (RD6) as inhibitors of ALKBH5 has also been recognized. These compounds, including leukemia and glioblastoma, demonstrate notable inhibitory effects on cancer cell lines. Studies have revealed that these compounds can reduce cell viability from 100 to 40% at low micromolar concentrations. Moreover, they exhibit a significant impact on inhibiting the activity of ALKBH5 within cells [127].DO-2728a is a novel and selective ALKBH5 inhibitor identified through structure-based virtual screening and optimization techniques. Compared to 2-oxoglutarate analogs, DO-2728a selectively targets ALKBH5, inhibiting its demethylase activity towards FTO. Experimental evidence suggests that DO-2728a effectively enhances m6A modification abundance in AML cells, diminishes the stability of TACC3 mRNA, and impedes cell cycle progression. Moreover, in a xenograft mouse model utilizing MV4-11 cells, DO-2728a significantly inhibits tumor growth while maintaining favorable safety profiles [128]. Experimental studies have shown that these inhibitors can effectively suppress ALKBH5 activity in both in vitro and in vivo models, thereby affecting m6A-modified RNA levels. Notably, in cancer research, ALKBH5 inhibitors have demonstrated significant anti-tumor potential by promoting apoptosis and inhibiting the proliferation of tumor cells. With further optimization and preclinical studies, these compounds are expected to become new cancer therapies.

## Conclusions

The m^6^A modification, a prevalent RNA alteration, is crucial in regulating the cell cycle and determining cell fate. Despite both belonging to the α-ketoglutarate (2OG)-dependent demethylase family, FTO, and ALKBH5 exhibit differences in intracellular functions and biological roles, likely stemming from variances in structure, substrate selection, cellular localization, and protein interactions. Notably, FTO and ALKBH5 may exert opposing effects within tumor contexts, regulated by factors such as tumor type, microenvironment, and cellular status. Moreover, research on inhibitors targeting FTO and ALKBH5 is advancing, with diverse inhibitors identified through high-throughput screening and structural optimization, demonstrating significance in targeting tumors, obesity, and other diseases.

This review comprehensively outlines the structure and function of the known m6A demethylation enzymes FTO and ALKBH5, elucidating their mechanisms in disease regulation. We delve into their intracellular localization, catalytic mechanisms, and interactions with other biological processes, focusing on their roles in tumor development, metabolic disorders, and neurological conditions. This underscores their significance in disease onset and progression. Furthermore, we discuss factors influencing FTO and ALKBH5 expression alongside recent developments in inhibitor research targeting them. Collectively, these studies furnish essential insights into the functions of m6A demethylases and their disease implications, offering new avenues for disease treatment and drug development in the future.

## Data Availability

Not applicable.

## Abbreviations

m^6^A: N6-methyladenosine
METTL3: Methyltransferase like-3
METTL14: Methyltransferase like-14
METTL4: Methyltransferase like-4
METTL5: Methyltransferase like-5
WTAP: Wilms tumor 1-associating protein
VIRMA: Viral-like m6A methyltransferase associated
RBM15: RNA binding motif protein 15
FTO: Fat mass and obesity-related protein
ALKBH5: Alkb homolog 5
YTHDC1-2: YTH Domain Containing 1–2
YTHDF1-3: YTH N6-Methyladenosine RNA Binding Protein 1–3
IGF2BP1-3: Insulin growth factor-2 mRNA binding protein 1–3
XPO2: Exportin 2
ssRNA: Single-stranded RNA
lncRNAs: Long non-coding RNAs
FOXO3a: Forkhead box protein O3
RUNX1T1: RUNX1 partner transcriptional co-repressor 1
FOXO1: Forkhead box O1
NAFLD: Non-alcoholic fatty liver disease
SREBF1: Sterol regulatory element binding Transcription Factor 1
STAT3: Signal transducer and activator of transcription 3
CSCC: Cervical squamous cell carcinoma
AML: Acute myeloid leukemia
ESCC: Esophageal squamous cell carcinoma
EC: Endometrial cancer
MTA1: Metastasis-associated protein 1
PDAC: Pancreatic ductal adenocarcinoma
GBM: Glioblastoma
HNSC: Head and neck squamous cell carcinoma
pNENs: Pancreatic neuroendocrine tumor
PC: Prostate cancer
CSC: Cancer stem cell
CNS: Central nervous system
Htr3a: 5-Hydroxytryptamine Receptor 3 A
EVs: Extracellular vesicles
ITGB1: Integrin beta-1
CXCL2: C-X-C Motif Chemokine 2
RBM33: RNA binding motif protein 33
Zfp217: Zinc finger protein 217
ISR: Integrative stress response
MA: Meclofenamic acid
MA2: Mefenamic acid’s ethyl ester
2OG: 2-oxoglutarate
snRNA: Small nuclear RNA
tRNA: Transfer RNA
EMT: Epithelial-mesenchymal transition
